# Self-Management and Its Predictors in adult Patients with Epilepsy: A Cross-Sectional Study

**DOI:** 10.4314/ejhs.v33i3.14

**Published:** 2023-05

**Authors:** Keivan Babaei, Mahnaz Khatiban, Mojtaba Khazaei, Leili Tapak, Farshid Shamsaei

**Affiliations:** 1 Ph.D Candidate, School of Nursing and Midwifery, Hamadan University of Medical Sciences, Hamadan, Iran; 2 Mother and Child Care Research Center, Department of Ethics Education in Medical Sciences, Department of Medical Surgical Nursing, School of Nursing and Midwifery, Hamadan University of Medical Sciences, Hamadan, Iran; 3 Department of Neurology, School of Medicine, Hamadan University of Medical Sciences, Hamadan, Iran; 4 Department of Biostatistics, School of Public Health and Modeling of Noncommunicable Diseases Research Center, Hamadan University of Medical Sciences, Hamadan, Iran; 5 Behavioral Disorders and Substance Abuse Research Center, Department of Nursing, School of Nursing and Midwifery, Hamadan University of Medical Sciences, Hamadan, Iran

**Keywords:** Self-management, Epilepsy, Seizures, Predictors

## Abstract

**Background:**

Appropriate self-management (SM) is crucial in controlling epilepsy and improving self-efficacy, medication adherence and avoidance of seizure triggers in patients with epilepsy (PWE. The aim of this study was to evaluate SM and its predictors among adult PWE in Iran.

**Methods:**

This cross-sectional study was conducted in 2021. The participants were 335 adult PWEs that were selected from healthcare settings in Hamadan, Iran. Epilepsy Self-Management Scale (ESMS) was used to measure patients' self-management behaviors. Data were analyzed using the independent samples t-test, Pearson's and Spearman's correlation analyses and multiple linear regression analysis.

**Results:**

The mean score of SM was 114.37±11, indicating moderate level of SM, and the mean scores of SM in the medication management, seizure management, and safety management subscales were significantly more than the mean scores of the other subscales (P < 0.05). Age, place of residence, marital status, seizure type, seizure frequency, and epilepsy duration significantly predicted 53% of the variance of SM (adjusted R square = 0.532).

**Conclusion:**

PWEs have moderate SM and need SM-related education, particularly about lifestyle management and information management. Predictors of SM should be considered to boost SM practice.

## Introduction

Epilepsy is a chronic neurologic condition characterized by frequent seizures. According to the World Health Organization, 50 million people in the world currently suffer from epilepsy, and 2.4 million people are diagnosed with epilepsy each year. The prevalence of epilepsy is 5–10 cases per 1000 people (1, 2). Patients with epilepsy (PWE) and uncontrolled seizures suffer from different problems. These patients may suffer from cognitive and learning impairment due to the involvement of the temporal lobe of the brain, frequent seizures, and medication side effects (3). Poor management of seizures, particularly generalized tonic-clonic seizures, also put PWEs at great risk for sudden unexpected death in epilepsy (4).

Anxiety disorders, sleep problems, and chronic fatigue among PWEs are also more prevalent than others (5). Besides direct epilepsy-related problems, PWEs suffer from different psychosocial problems that affect their lives, including depression, social isolation, sense of guilt and embarrassment, low self-confidence, pessimism towards life, social stigmatization, and suicide. These problems significantly contribute to their chronic disability and reduce their quality of life (6). The multiplicity of epilepsy-related problems among PWE highlights the great importance of effective epilepsy management.

Anti-seizure medications remain the mainstay of the treatment of epilepsy (7). Nonetheless, around 40% of PWEs who receive antiepileptic medications suffer from frequent seizures. Evidence shows that effective seizure management and quality of life improvement among PWEs need a multi-component approach that relies not only on drug therapy, but also on effective self-management (SM) (8). SM of epilepsy is defined as the steps a person takes to control seizures and to control the effects of having a seizure disorder. Patient SM behaviors are important in controlling epilepsy, because decreased patient participation in treatment regimens is a major cause of breakthrough seizures, leading to increased hospital use and mortality (9). The chronic course of epilepsy and the inability of healthcare providers to effectively fulfill the psychosocial needs of PWEs due to the lack of an effective interdisciplinary approach and time limitation also highlight the necessity of effective SM for effective epilepsy management (10).

SM in epilepsy has different dimensions. A study reported that the five dimensions of SM in epilepsy are medication management, seizure management, information management, safety management, and lifestyle management (11). Another study reported that the eleven epilepsy management skills are coping, healthcare communication, medication adherence, pro-activity, safety, seizure tracking, seizure response, social support, stress management, treatment management, and wellness (12). Several studies have shown that effective SM had many different outcomes. A study reported that SM interventions led by epilepsy nurses reduced the costs of hospitalization in emergency wards by £558 per year and lowered the rate of emergency ward attendance by 55% (8). Another study revealed that SMS-based SM education for PWEs improved the different aspects of their quality of life, especially seizure worry, emotional well-being, and social functioning (13). Robust SM skills in PWEs may improve their self-efficacy and medication adherence, prevent seizure triggers, and increase patient and family knowledge regarding when to seek urgent medical care (8–9).

Previous studies reported contradictory results regarding SM among PWE. A study on 172 immigrant PWE in the United States indicated that the mean score of SM in these patients was 144 in the possible range of 38–190 and the lowest and the highest dimensional mean scores were for the information management and the medication management subscales, respectively (14). The mean score of SM in another study on fifty PWEs was 141, and the lowest and the highest dimensional mean scores were for the lifestyle management and the medication management subscales, respectively (15). Another study found that the highest SM dimensional mean score was for the pro-activity, medication adherence, and healthcare communication subscales, while the lowest dimensional mean score was for the stress management and the safety management subscales (16). Studies into the contributing factors of SM among PWE also reported contradictory results. A study found that age and number of medications had significant positive correlations with SM, while gender, educational level, and number of seizures had no significant correlations with SM (17). However, a study reported that married patients, employed patients, and patients with higher educational level had lower SM, and that there was a significant positive correlation between SM and seizure frequency (15, 17).

The contradictory results of previous studies into SM and its contributing factors among PWEs highlight the need for further studies in this area. Moreover, SM is a context-bound concept (18) and hence, studies in different contexts are needed to provide more conclusive evidence about it. Therefore, the present study was conducted aiming to evaluate SM and its predictors among adult PWEs in Iran.

## Methods

**Design**: This cross-sectional study was conducted in 2021. The Strengthening the Reporting of Observational Studies in Epidemiology (STROBE) checklist was used to report important aspects of this study.

**Setting and participants**: Study setting was Hamadan Epilepsy Association and the neurology care wards of Be'sat and Sina hospitals in Hamadan, west of Iran. Adult PWE who referred to the study setting constituted the study population. Eligible PWE were selected through convenience sampling. The eligibility criteria were age over 19 years, treatment with at least one antiepileptic drug for at least 1 year, definite diagnosis of epilepsy by a neurologist according to the criteria of the International League Against Epilepsy (19), willingness to complete self-reported questionnaires, no history of mental or psychological disorders, and basic literacy skills. Reluctance to stay in the study, intellectual disability or neurological deficits that affect daily living activities and having major medical, surgical, or psychiatric illnesses were the exclusion criteria.

**Sample size estimation**: Sample size was calculated with a confidence level of 95%, a power of 80%, and a *d* of 2 and using the results of a study on PWE which reported that the standard deviation of SM was 17.81 (20). The output of the sample size calculation formula *n* = (*Z*^2^_1−β/2_σ^2^)/*d*^2^ showed that at least 305 participants were needed. Given the probability of participants' withdrawal from the study, sample size was increased to 335.

**Data collection**: Data were collected using a demographic questionnaire, a clinical characteristics questionnaire, and the Epilepsy Self-Management Scale (ESMS). The items of the demographic questionnaire were on age, gender, marital status, educational level, and place of residence and the items of the clinical characteristics questionnaire were on epilepsy duration, number of seizures in the past three months, and seizure type.

The ESMS, developed by Dilorio et al., was used for SM assessment. ESMS is a commonly-used and well-validated self-reported scale that assesses the frequency with which individuals perform tasks that are helpful in managing their seizures. This scale has 38 items on SM behaviors among PWE in five subscales; namely, information management (eight items), medication management (ten items), seizure management (six items), safety management (eight items), and lifestyle management (six items). Items are scored on a five-point scale as follows: 1: “Never”; 2: “Rarely”; 3: “Sometimes”; 4: “Most of the times”; 5: “Always”. Thus, the possible total scores of the scale and its five subscales are, respectively, 38-190, 8–40, 10–50, 6–30, 8–40, and 6–30, with higher scores showing higher SM. A study reported that this scale has acceptable content validity (with a content validity index of 0.93) and acceptable reliability (with Cronbach's alpha values of 0.81–0.86) (21). The scores of the scale and its subscales were normalized and changed into the 0–100 scale. Participants completed the study instruments through the self-report method. The validity and reliability of the Persian version of ESMS for a sample of Iranian patients with epilepsy was previously evaluated and confirmed (22).

**Statistical analysis**: Statistical Package for the Social Sciences (SPSS) software package (version 21.0, SPSS Inc., Chicago, IL, USA) was used for data analysis. Normality of the data was evaluated using the Kolmogorov-Smirnov test. Mean and standard deviation (Mean±SD) were calculated for the scores of SM and its subscales. The relationship of SM with dichotomous demographic and clinical characteristics was tested through the independent samples *t*-test and its correlation with age, educational level, number of seizures, and epilepsy duration was tested through Pearson's and the Spearman's correlation analyses. The predictors of SM were determined using multiple linear regression analysis. P-value less than 0.05 was considered statistically significant.

**Ethical considerations**: This study has the approvals of the Institutional Review Board (code: 140007075998) and the Ethics Committee (code: IR.UMSHA.REC.1400.322) of Hamadan University of Medical Sciences, Hamadan, Iran. Participants were provided with explanations about the aim of the study and data confidentiality and informed consent was obtained from all of them.

## Results

All 335 participants completed the study. The mean of their age was 29 years (range: 19–55). One-third of the participants had primary education (33.4%) and most of them were females (50.4%), employed (55.2%) and married (55.5%), lived in urban areas (61.8%), and suffered from tonic-clonic seizures (77.9%). More than one-third of them reported no history of seizure in the past three months (37.3%). The mean of epilepsy duration was sixteen years in the range of 2–50.

The normalized mean score of SM was 50.2±7.8 in total, 65.91±8.3 in the medication management subscale, 29.01±8.4 in the information management subscale, 53.91±10 in the seizure management subscale, 32.2±6.1 in the lifestyle management subscale, and 62.39±7 in the safety management subscale (Table 1).

**Table 1:** The mean scores of self-management and its subscales

Self-management	Number of items	Score range	Normalized Mean±SD
Medication	10	10–50	65.91±8.3
Safety	8	8–40	62.39±7
Information	8	8–40	29.01±8.4
Seizure	8	6-30	53.91±10
Lifestyle	6	6-30	32.2±6.1
Total	38	38-190	50.2±7.8

The results of the independent samples *t*-test revealed no significant difference between male and female participants and between participants who lived in urban and rural areas respecting the mean score of SM (P>0.05). However, the mean score of SM among unemployed participants, single participants, and participants with tonic-clonic seizures was significantly greater than employed participants, married participants, and participants with other seizure types, respectively (P<0.05). The results of correlation analysis also showed that SM had a significant positive correlation with age (r = 0.124; P = 0.024), educational level (r = 0.508; P < 0.001), seizure frequency (r = 0.626; P < 0.001), and epilepsy duration (r = 0.574; P<0.001) (Tables 2 and 3).

**Table 2:** The relationship of self-management with demographic and clinical characteristics

Characteristics		N (%) or Mean±SD	Mean±SD	r	*P*-value
Gender	Male	166 (49.6)	115.51±22.06	0.513*	0.349
	Female	169 (50.4)	113.27±21.76		
Employment status	Employed	185 (55.2)	110.39±20.66	5.854*	<0.001
	Unemployed	150 (44.8)	119.30±22.47		
Marital status	Single	149 (44.5)	118.07±23.58	3.839*	0.007
	Married	186 (55.5)	111.42±20.05		
Place of residence	Rural areas	128 (38.2)	113.40±21.9	0.026*	0.300
	Urban areas	207 (61.8)	115.96±21.8		
Seizure type	Tonic-clonic	261 (77.9)	118.08±22.77	33.533*	<0.001
	Other	74 (22.1)	101.31±11.33		

* The results of the independent-sample *t* test

**Table 3:** The relationship of self-management with demographic and clinical characteristics

Characteristics		N (%) or Mean±SD	r	*P*-value
Educational level	Basic	48 (14.3)	0.508^	<0.001
	Primary	112 (33.4)		
	Guidance school	93 (27.8)		
	High school	62 (18.5)		
	University	20 (6)		
Seizure frequency	0	125 (37.3)	0.626^	<0.001
	1	114 (34)		
	2	57 (17)		
	≥ 3	39 (11.6)		
Age (Years)		29±8.2	0.124#	0.024
Epilepsy duration		16±1.1	0.574#	<0.001

^ The results of the Spearman's correlation analysis

# The results of the Pearson's correlation analysis

The results of the multiple linear regression analysis indicated that age, place of residence, marital status, seizure type, seizure frequency, and epilepsy duration significantly predicted 53% of the variance of SM (adjusted R square = 0.532; Table 4).

**Table 4:** The results of regression analysis to determine the predictors of self-management

Variables		B	Std. Error	Beta	*t*	*P*-value
Constant		92.042	16.880		5.453	<0.001
Age		0.526	0.171	0.198	3.082	0.002
Gender	Female (Reference)	—	—	—	—	—
	Male	0.541	2.658	0.012	0.204	0.839
Epilepsy duration		0.743	.149	0.392	4.983	<0.001
Seizure frequency		3.854	1.566	0.179	2.461	0.014
Seizure type	Other (Reference)	—	—	—	—	—
	Tonic-clonic	15.486	3.331	0.294	4.650	<0.001
Place of residence	Rural (Reference)	—	—	—	—	—
	Urban	4.575	2.089	0.099	2.190	0.029
Marital status	Married (Reference)	—	—	—	—	—
	Single	8.742	3.677	0.101	2.378	0.018
Employment status	Employed	—	—	—	—	—
	Unemployed	2.838	3.051	0.064	0.930	0.353
Educational level	Basic (Reference)	—	—	—	—	—
	Primary	13.669	15.818	0.243	0.864	0.388
	Guidance school	10.354	15.566	0.211	0.665	0.506
	High school	17.837	15.568	0.382	1.146	0.253
	University	24.191	15.735	0.397	1.536	0.126

R square = 0.560

Adjusted R square = 0.532

Confounding factors: Age; Gender; Marital status

## Discussion

This study aimed to evaluate SM and its predictors among adult PWEs in Iran. The findings showed that the mean scores of SM in the medication management, seizure management, and safety management subscales were significantly better than the mean scores of the information management and lifestyle management subscales. Contrary to our findings, a study in the United States reported that the total mean score of SM was more than the mean score in our study. This contradiction may be attributable to the fact that SM is a context-bound concept influenced by attitudes, beliefs, emotions, and socio-cultural and financial resources (23).

Assessment of Iranian's lifestyle showed that modernization, technological advances, and urbanization have been associated with the prevalence of unhealthy lifestyle habits such as immobility and consumption of fast foods and have increased the level of perceived stress in life in Iran (24). The results of a study showed that policy makers' poor planning for public life, limited attention to public health education, inadequacy of educational materials for health promotion and lifestyle modification in schoolbooks, and families' inattention to healthy lifestyle have reduced non-adherence to traditional healthy lifestyle behaviors (25).

Our findings also showed that PWEs had poor adherence to healthy eating recommendations. A meta-analysis on nineteen studies with a total sample of 1084 child PWE also showed that the use of ketogenic diet helped completely manage seizure in 16% of cases, reduced seizure by 90% in 32% of cases, and reduced seizure by 50% in 50% of cases (26). Therefore, PWEs should receive appropriate education about this type of diet.

We also found that PWEs had limited physical activity. A systematic review showed that limited physical activity is associated with comorbidities and reduces quality of life among PWEs (27). Our findings also indicated poor stress management among PWEs. This finding may be due to social stigmatization, verbal mockery, prejudice, fear of disease disclosure, and sense of loss among these patients (28). Study findings also showed that PWEs had problems in effective sleep management. This problem is attributable to factors such as epilepsy-related stress and anxiety, nighttime seizure, use of antiepileptic medications, and use of high doses of sedatives which can lead to sleep resistance over time (29). A study showed that 1.5-hour sleep deprivation was associated with obvious increase in the frequency and duration of early myoclonic seizures during the first two hours after wake-up among adolescent PWEs (30). Another study indicated that getting sufficient sleep is a modifiable risk factor for better SM in PWE (31).

We also found that the lowest SM dimensional mean score was for the information management subscale. The items of this subscale are on the documentation of seizure type and frequency, awareness of the side effects of antiepileptic medications, participation in counseling and educational programs, and family awareness of the appropriate measures during seizures (11). The low score of this subscale may be due to participants' limited knowledge. The results of a study showed that lack of epilepsy knowledge is a community and family barrier for SM in PWE (32).

Study findings also indicated that SM had a significant positive correlation with age so that older participants had better SM. This is in agreement with the findings of two previous studies (15, 33). SM in the present study also had a significant positive correlation with educational level, which is consistent with the findings of a previous study (34). Another study on patients with chronic diseases showed that patients with higher educational level valued these behaviors more and had better understanding about the effects of healthy eating and physical activity than patients with lower educational level (35).

Another finding of the present study was that employed PWEs obtained significantly lower SM than their unemployed counterparts, which is in agreement with the findings of a previous study (16). Employed patients are at risk for social stigmatization at workplace (36), which is associated with discrimination and rejection at workplace (37). PWEs experience continuous fear and concern at workplace due to the unpredictability of their seizures and their lack of control over them, and stigmatization causes them greater stress and mental strain (38). Our findings also showed that SM among married PWEs was lower than their single counterparts. A previous study also reported the same finding (1). Another study showed that the prevalence of comorbid psychiatric conditions such as anxiety and depression among married PWEs was higher than among single PWEs (39).

We also found that SM had a significant positive correlation with the seizure frequency, which is consistent with the findings of a previous study (17). More seizures are associated with greater hospital attendance of PWEs, greater disability in performing social roles, more absences from work and education, and greater likelihood of social stigmatization, which in turn reduce their quality of life and hope (40). Therefore, PWEs who experience more seizures are more likely to have greater adherence to SM behaviors such as regular medication intake, adequate sleeping, low stress lifestyle, and avoidance from seizure risk factors such as alcohol consumption.

In conclusion, this study showed that PWEs had moderate adherence to SM behaviors, and that the significant predictors of their SM were age, place of residence, marital status, seizure type and frequency, and epilepsy duration so that patients with older age, residence in urban areas, bachelorhood, affliction by tonic-clonic seizures, greater seizure frequency, and longer epilepsy duration. Therefore, PWEs need SM-related education, particularly about lifestyle management (i.e., healthy eating, regular physical activity, adequate sleep, and stress management) and information management (i.e., documentation of seizure type and number, antiepileptic medication side effects, and necessity to participate in group counseling programs). Predictors of SM should be considered to boost SM practice.
